# Initial Characterization of Morpho-Anatomical Traits and Antioxidant Profile of *Iris brandzae* Prodan from Romania’s Wild Flora Under Culture Conditions

**DOI:** 10.3390/plants14243803

**Published:** 2025-12-13

**Authors:** Lucia Draghia, Maria Apostol, Culiță Sîrbu, Ivayla Dincheva, Maria Daniela Mihăilă Ionică, Rodica Mihaela Dinică, Mariana Lupoae, Raluca-Maria Hlihor, Isabela Maria Simion, Ciprian Chiruță, Diana Elena Bolohan, Jose Reig Arminana, Francisco José Garcia Breijo

**Affiliations:** 1 “Ion Ionescu de la Brad” Iasi University of Life Sciences, 3 Mihail Sadoveanu Alley, 700490 Iasi, Romania; lucia.draghia@iuls.ro (L.D.); culita.sirbu@iuls.ro (C.S.); raluca.hlihor@iuls.ro (R.-M.H.); isabela.simion@iuls.ro (I.M.S.); ciprian.chiruta@iuls.ro (C.C.); diana.bolohan@iuls.ro (D.E.B.); 2 Department of Agrobiotechnologies, Agrobioinstitute, Agricultural Academy, 8 Dragan Tsankov Blvd, 1164 Sofia, Bulgaria; ivadincheva@yahoo.com; 3 Department of Chemistry, Physics and Environment, Faculty of Sciences and Environment, “Dunarea de Jos” University of Galati, 111 Domneasca Street, 800201 Galati, Romania; maria.mihaila@ugal.ro (M.D.M.I.); rodinica@ugal.ro (R.M.D.); 4 Department of Pharmaceutical Sciences, Faculty of Medicine and Pharmacy, “Dunarea de Jos” University of Galati, 35 Alexandru Ioan Cuza Street, 800010 Galati, Romania; mariana.lupoae@ugal.ro; 5 Medical-Pharmaceutical Research Center, Faculty of Medicine and Pharmacy, “Dunarea de Jos” University of Galati, 35 Alexandru Ioan Cuza Street, 800010 Galati, Romania; 6 Instituto Cavanilles de Biodiversidad y Biología Evolutiva, Universitad de València, 46980 Valencia, Spain; jose.reig@uv.es; 7 Departamento de Ecosistemas Agroforestales, Escuela Técnica Superior de Ingeniería Agronómica y del Medio Natural (ETSIAMN), Universitat Politècnica de València, Camino de Vera s/n., 46022 Valencia, Spain

**Keywords:** *Iris brandzae*, chlorophyll, vascular bundles, flavonoid content, phenolic content

## Abstract

In Romania’s wild flora, several *Iris* species exhibit important ornamental characteristics, such as early spring flowering and resilience to abiotic stress. This study assessed the behavior to new ecological conditions, the ornamental potential, and the antioxidant capacity of the wild species of *Iris brandzae* using morpho-anatomical, physiological, and biochemical biomarkers. The study of phenotypic characteristics (number and size of leaves on sterile and fertile shoots, size of flowering stems, bracts protecting the flowers, and perianth-segments) aimed to confirm and supplement existing information in the literature, as well as to evaluate the ornamental potential of this species. Morphological analyses revealed clear differences between fertile and sterile shoots, while photosynthetic activity across phenophases showed values within normal parameters, with the maximum recorded during flowering and with the chlorophyll *a*/chlorophyll *b* ratio maintained at values close to 3:1, indicating favorable cultivation conditions. Biochemical investigations (total phenolic content (TPC), total flavonoid content (TFC), and antioxidant activity) demonstrated that dried plant material, particularly roots, contained higher levels of phenolic and flavonoid compounds and exhibited stronger antioxidant activity compared to fresh material. By integrating morpho-anatomical, physiological, and biochemical data, this research provides the first comprehensive characterization of *I. brandzae* beyond taxonomic and ecological descriptions. Our findings emphasize the species behavior under cultivation conditions, its ornamental value, and its potential as a source of bioactive compounds relevant to pharmaceutical applications.

## 1. Introduction

The genus *Iris* (Iridaceae Juss.) comprises 200–400 species distributed across the Northern Hemisphere, mainly in Eurasia, North America, and Africa [1,2,3,4]. The vast natural range of the genus, encompassing diverse ecological conditions (arid or rocky terrain, swamps or floodplains, meadows, grassland or mountainous areas), has contributed to a wide diversity of ecological, morpho-anatomical, cytological, and biomolecular characteristics in *Iris* species. This diversity has often complicated the study of the evolutionary and systematic relationships within the genus [5,6,7]. Over time, the systematics of the genus *Iris* and the classification of its taxa have generally been based on morpho-anatomical (especially of flowers and underground organs), ecological characteristics as well as on cytogenetic analyses [4,6].

The rusticity of irises and their ability to adapt to the most diverse conditions have led to many of the wild species being brought into cultivation as ornamental plants. In Romania, the most popular is *I. germanica*, found in most gardens, but other species may also be present, such as *I. pumila*, *I. pseudacorus*, and *I. sibirica*, mentioned in the specialized literature [8]. Also, wild *Iris* species from the Romanian flora are considered very valuable for ornamental plant breeding [9].

The Romanian flora includes 15 naturalized and native *Iris* species [9,10], four of which are classified in the sozological category ‘Vulnerable’—VU [11]: *I. aphylla* L., *I. brandzae* Prodan, *I. pontica* Zapał., and *I. humilis* Giorgi. *I. brandzae* has a rather limited natural range, fragmented in populations occupying different habitats, located in mainly in Romania and, to a lesser extent, in the Republic of Moldova and Ukraine [12,13]. Documented sites include the meadows of the Prut River in the Republic of Moldova [14], in the Hotin–Mohyli region of Transnistria, in some parts of Ukraine and northern Republic of Moldova [15], or in other areas in Ukraine [16,17,18].

*I. brandzae* has been reported in Romania in the northern, northeastern, and southeastern regions, more specifically in the historical provinces of Moldova (Botoșani, Iași, Vaslui, Vrancea, Galați counties) and Wallachia (Buzău and Prahova counties). Synthesis studies [11,13,19] documented populations across several localities, while subsequent research confirmed many of these sites and added new ones, including areas in Buzău, Vaslui, and Iași counties [20,21,22]. Additional records derived from the herbarium vascular plants collection of ‘Alexandru Ioan Cuza’ University in Iași, Romania, expanded the distribution to further sites in Botoșani, Iași, Vaslui, and Buzău counties [23].

In the Romanian flora, *I. brandzae* is considered a subendemic species [11,13,24] and its sozologic classification varies depending on the authors: Vulnerable—VU [11,24,25] or Low risk—LR [24]. In terms of spread, some authors consider it rare [20,22,24] or relatively rare [23]. More recent research indicates a decrease in *I. brandzae* populations, as a result of the reduction in grassland areas that constitute their natural habitats, such that it is considered one of the most threatened plant species in Romania [13,26]. In Ukraine it is included in the endangered (EN) species category, with scarce occurrence (spread) [15,27].

The species is adapted to the temperate continental climate, with cold winters and hot, dry summers. It has a fairly high ecological plasticity, being found in hayfields, pastures, or meadows at altitudes ranging from 20 m to almost 300 m, with sunny exposure or on semi-shaded slopes [13]. Although it prefers well-drained soils, rich in humus and with a pH close to neutral [13], it also grows on wet or heavily degraded and saline soils, on leached chernozems and forest ashes [20,21]. Phytocoenological studies conducted in habitats in Romania revealed a set of phytogeographic characteristics and floristic compositions specific to the populations of *I. brandzae* identified in Wallachia and Moldova. According to recent data published by Chirilă et al. [13], *I. brandzae* has been recorded in a wide range of plant communities, both xero-mesophilic (class Festuco-Brometea Br.-Bl. et Tx. ex Soó 1947), and mesophilic (class Molinio-Arrhenatheretea Tx. 1937) and even ± halophilic (class Festuco-Puccinellietea Soó ex Vicherek 1973), but most often, it occurs in xero-mesophilic meadows of the association Taraxaco serotinae-Festucetum valesiacae [28,29,30].

The *Iris* genus is a valuable source of various bioactive compounds with significant pharmaceutical relevance and has long been appreciated for its medicinal, cosmetic, food, dyeing, and other applications [3,31,32,33,34,35]. Numerous species contain flavonoids (apigenin, quercetin, irigenin, tectoridin), phenolic acids, xanthones, and essential oils, which exhibit antioxidant, anti-inflammatory, antimicrobial, antitumor, and neuroprotective properties. Isoflavones and their glycosides isolated from rhizomes of *I. germanica*, *I. pallida*, and *I. ensata* demonstrated cytotoxic and estrogenic activities, suggesting their potential in cancer- and hormone-related therapies [3,36]. Extracts from *I. pseudacorus* and *I. sibirica* showed significant antioxidant capacity and inhibitory effects on lipid peroxidation and enzyme activity related to inflammation [37].

Beyond modern pharmacology, the *Iris* genus has a long history of use in traditional medicine as an astringent, diuretic, and tonic agent [3]. Species such as *I. germanica*, *I. albicans,* and *I*. *adriatica* have been shown to contain flavonoids, isoflavonoids, and xanthones such as naringenin, genistein, irigenin, iridin, tectoridin, and mangiferin with antioxidant, anti-inflammatory, antimicrobial activity and chemoprotective potential [35,38]. Jaegerova et al. [39] identified, for the first time, in *Iris versicolor* p-coumaric acid, caffeic acid, chlorogenic acid, which are phenolic acids, and also terpenoids such as iridal, iritectol, and iridogermanal, studied for their cytotoxicity and chemopreventive activities. Khatib et al. [3] published a report discussing the chemical composition of essential oils obtained from *Iris* spp. and their benefits. In the study conducted by Abdel-Baki et al. [40], the high antiplasmodial and antileshmanial potentials of the *I. confusa* extract were highlighted in relation to its metabolic profile, which contains glycolipids, triacyl glycerols, iridals, and oleic acid.

The consumption of ornamental plants reflects the level of civilization and well-being, fulfilling multiple functions such as interior and landscape decoration, air purification, mental health improvement, and economic value. Rising incomes and urbanization have fueled global demand, with international statistics projecting a growth in the global flower market from USD 63.49 billion in 2025 to USD 115.86 billion in 2035. Although cut flowers dominate in value, including in the perspective of the next 10 years (from USD 18.5 billion in 2025 to USD 32 billion in 2035), a significant development of the segment of potted plants and garden plants is also expected, driven by interest in gardening and sustainable living practices, highlighting the importance of valorizing native and wild ornamental species [41].

The present study aimed to evaluate the behavior of *I. brandzae* to cultivation technology and its ability to preserve the ornamental characteristics, with the goal of recommending its inclusion in the assortment of valuable ornamental plants, as well as to identify phytochemical compounds with antioxidant properties. The literature confirms that *Iris* species possess complex secondary metabolites (polyphenols, flavonoids, anthocyanins, terpenoids), their composition being variable depending on species, area, cultivation conditions, etc. The *Iris* species compounds have also been identified to have tolerable toxicological properties, and so, they can be promising phytopharmaceuticals [3,42]. Given the continuous need for natural compounds with antioxidant, antibacterial, and cytotoxic properties, the study of the chemical composition and biological properties of *Iris* species is of great interest [35,43].

The results presented in this paper serve to complete the information on *I. brandzae*, considering that most studies found in the literature mainly refer to the botanical description, distribution, ecology, and biogeography of the species [1,2,44] or to some aspects regarding the karyotype, presence in botanical garden collections, ultrastructure of the seeds surface, and importance as a source of germplasm in breeding work [9,45,46]. From our knowledge, anatomical, physiological, and biochemical studies are lacking. To address this gap, our research focused on the following directions: (i) statistical analysis of ornamental morphological characters of the *I. brandzae* species; (ii) assessment of the photosynthetic pigment content providing information on the physiological response to cultivation; (iii) examination of the anatomical structure of the leaf to complement physiological and biochemical studies; and (iv) determination of antioxidant compounds, highlighting the dual value of *I. brandzae*, as an ornamental species and a potential source of bioactive metabolites for pharmaceutical applications.

By integrating morphological, physiological, anatomical, and biochemical analyses with statistical analysis, our research advances the characterization of *I. brandzae* and supports its potential valorization in ornamental breeding and phytochemical exploration.

## 2. Results

### 2.1. Species Description

*Iris brandzae* Prod. Bul. Grăd. Bot. Cluj, XV (1935), 103, tab. XIII (Syn.: *I. sintenisii* Janka subsp. *brandzae* (Prodan) Prodan; *I. sintenisii* Janka subsp. *brandzae* (Prodan) D.A. Webb & Chater, comb. superfl.).

Stems 15–25 cm, cylindrical to weakly compressed, not winged. Leaves 1.5–2.5 (3.5) mm wide, with 2–3 (5) prominent veins, scabrid, those on the flowering stem reaching or slightly exceeding the level of the flowers, those on sterile shoots much longer (up to 50 cm). Spathe 50–80 × 6–8 mm, strongly inflated, with prominent veins, scabrid. Flowers 2(1), not fragrant; ovary 12–15 mm long, 6-winged, with a slender beak, of 12–22 mm long; hypanthial tube 18–25 mm long; the outer perianth-segments un-bearded, patent to slightly deflexed, pale purplish, with dark purple veins, the claw 30–33 mm long, the limb 12 × 9–10 mm; the inner perianth-segments erect, purple, 42 × 7 mm; stamen 3; style branches purple. Capsule 1.5 cm long, 6-winged, with a very long beak.

The species *Iris brandzae* was first described by I. Prodan (1935/1936), from Romania, but the taxonomic rank of this plant is still disputed. Webb and Chater [47] changed the rank of this taxon to the position of subspecies (subsp. *brandzae*), and subordinated it to *I. sintenisii* Janka. Under the name *I. sintenisii* Janka subsp. *brandzae* (Prodan) D.A. Webb and Chater, this plant was subsequently listed both in Flora Europaea [48] and in some international floristic databases (e.g., [49,50]).

However, in all floristic syntheses of Romania (e.g., [10,51,52]), as well as in the *Red Book of Vascular Plants of Romania* [53], the taxon described by Prodan [54] is treated as a true species, which also corresponds to our opinion.

### 2.2. Morphological Characters

The morphological studies were aimed to evaluate the potential of the species to adapt to new growing conditions and to maintain ornamental characters during the study period. The analysis of phenotypic characteristics of *I. brandzae* species within the experimental field, was useful not only for confirming and supplementing existing information in the literature, but also for evaluating the ornamental potential of the species. Additional clarifications are even more justified given that *I. brandzae* has often been confused with *I. sintenisii* Janka. Indicative data on the morphometry of *I. brandzae* plants from populations identified in Romania have been reported only in botanical determinants that include brief descriptions of taxa. In general, the results obtained in the experimental field fall within the limits specified in the botanical diagnoses [55], with some minor differences attributable to the influence of pedoclimatic conditions, the floristic composition of the site in question, or other specific factors.

Since the species *I. brandzae* belongs to the category of plants that decorate not only by its flowers, but also by its beautiful foliage, the biometric determinations were made on both fertile and sterile shoots and included measurements for both morphological characters.

Leaf apparatus development was carried out for both sterile and fertile shoots and included determinations of leaf length and width. In the case of fertile shoots, in addition to the determinations on leaf growth, measurements were also made on the height of the flowering stem, the number of flowers per stem, and the length and width of the perianth-segments.

For the analysis and comparison of morphological parameters measured in *I. brandzae*, a linear regression model was used to investigate the relationship between two variables (e.g., leaf length and leaf width) [56]. In this way, it was possible to evaluate the influence of each parameter on the dependent variable selected in the study. The analysis of the length of the bracts (spathes) was performed using a *t*-test (Student’s), by comparing the mean values of two sets of biometric data, selected from the study, while the one-way ANOVA test was applied to determine the existence of significant differences between the average lengths of the bracts in the four independent data groups. In addition, multiple regression models were tested using flowering stem height as the dependent variable and bract length as independent variables, but they were not included in this study because they did not reveal any statistically significant relationships (*p* > 0.05).

The normality of data distribution was verified by the Kolmogorov–Smirnov test to validate the use of parametric tests The statistical analysis was performed in the Microsoft Excel application (Office Professional 2019 package), with a significance level set at 0.05.

Table 1 shows the average values for leaf length and width in sterile and fertile shoots, along with the confidence intervals for these averages. It was found that the leaves of sterile shoots are much longer than those of fertile shoots, with average values of 50.5 cm and 18.4 cm, respectively, which confirms the data in the literature. The differences recorded in width were smaller but still in favor of the leaves of sterile shoots.

**Figure 1:**
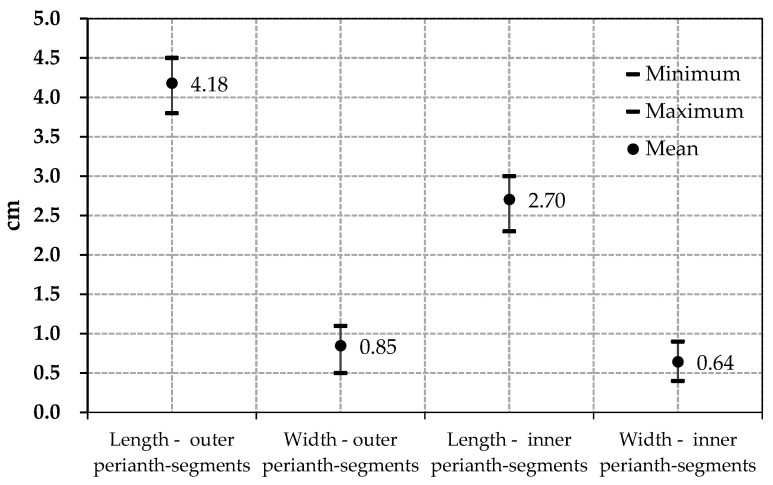
Intervals of variation in the values measured for the outer and inner perianth-segments.

In the same way, data on the height of the flowering stems and the dimensions of the perianth-segments are presented (Table 2). The mean value of the height of the flowering stems was 27.31 cm; values close to those mentioned in the botanical descriptors (approx. 23 cm). Also, according to data found in the literature, the perianth-segments are unequal, with the outer ones longer and wider and the inner ones shorter and narrower. The intervals of variation in the values of these characters are graphically represented in Figure 1.

**Figure 2:**
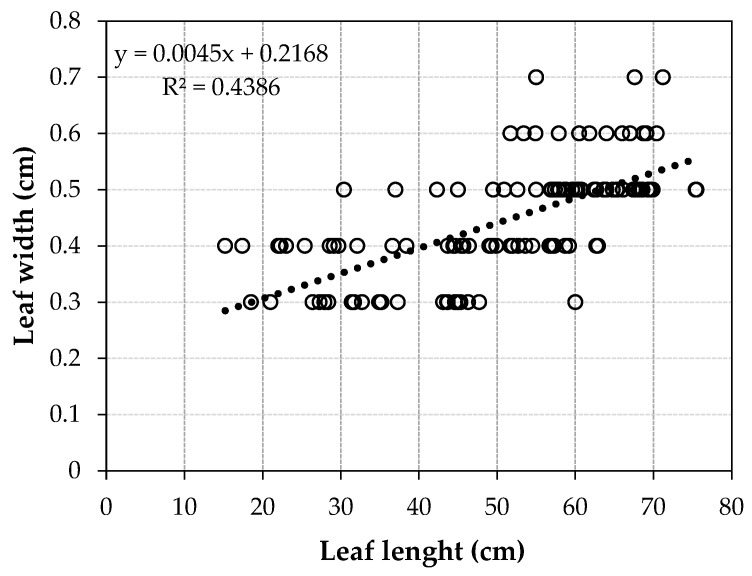
Linear regression model between leaf width and leaf length.

The Pearson correlation coefficient (r = 0.63, between leaf length and leaf width) indicates a moderate positive linear association, suggesting that, on average, longer leaves tend to be wider. The linear regression model obtained between leaf length and leaf width (y = 0.0045 + 0.2168) has a correlation coefficient of R^2^ = 0.4386, indicating that about 44% of the variation in leaf width can be explained by the variation in leaf length (Figure 2).

Another series of observations was represented by some characters of the fertile shoots (number of leaves/shoot, length of the four bracts). Botanical diagnoses indicate the presence of two well-developed leaves on fertile shoots, but determinations in the experimental field recorded a value of 2.38, within a confidence interval of 2.47–3.20. An additional analysis was performed on the bracts (spathes) that protect the flowers and any correlations with the length of the flower stalk. All plants in the study sample had four bracts/stem (two on each flower), numbered from the base of the stem to the tip (01, 02, 03, and 04). The data were collected in this order, forming four distinct groups from 25 different plants. Regarding the length of the bracts, it was found that the bracts located at the bottom of the flowers (01 and 03) were longer, with averages of 8.68 and 8.02 cm, respectively, compared to those protecting the flowers at the top (02 and 04), whose average length was 6.25–7.17 cm (Table 3).

Application of the one-way ANOVA test revealed significant differences (*p* = 2.681 × 10^−8^) in the mean bracts length in the four independent data groups (01, 02, 03, 04), representing the position of the bracts on the stem (Table 4). Verification of the results was performed using Student’s *t*-test for groups of two-by-two bracts; the *p*-values obtained are recorded in Table 5.

Student’s *t*-test shows significant differences between groups for bract length 01, 02 and 04 (*p* < 0.05), indicating a real variation between groups for these characteristics (Table 5). For bract length 01 and 03, the value *p* = 0.085 suggests that the means of these variables do not differ significantly, indicating similar bract length development.

### 2.3. Study of the Anatomical Structure of Leaves

**Figure 3:**
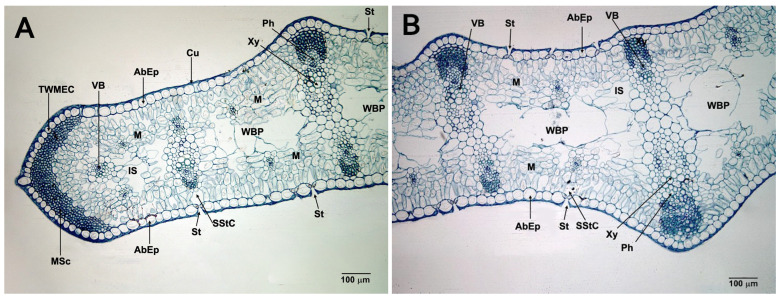

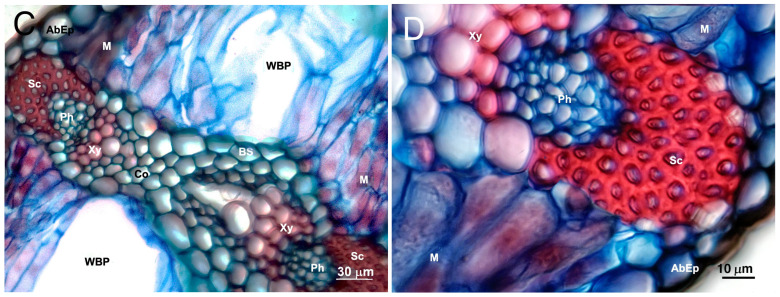
Cross-sections of *I. brandzae* L. leaf. Semi-thin sections made with a resin microtome and stained with toluidine blue: (**A**) general view of the leaf (×100); (**B**) detail of the leaf with two vascular bundles facing each other and joined by collenchyma (×100). Sections made with a freezing microtome and stained with FSA: (**C**) detailed vascular bundles facing each other and united by collenchyma (×400); (**D**) detail of a vascular bundle (×1000). Abbreviations: AbEp: Abaxial Epidermis; BS: Bundle Sheath; Co: Collenchyma; Cu: Cuticle; M: Mesophyll; Ph: Phloem; Sc: Sclerenchyma; TWMEC: Thick-Walled Marginal Epidermal Cell; WBP: Water-Bearing Parenchyma; Xy: Xylem.

**Figure 4:**
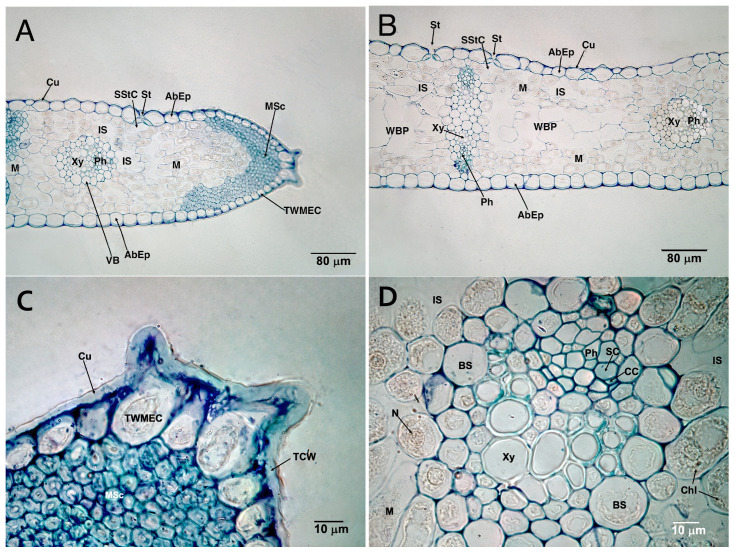

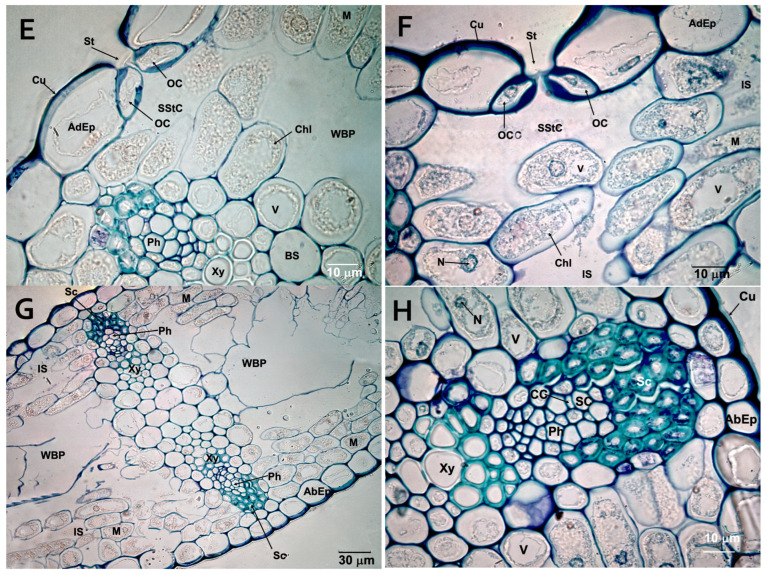
Cross-sections of *I. brandzae* L. leaf. Semi-thin sections were made with a resin microtome and stained with toluidine blue. (**A**) Detail of the end of the leaf blade (×200); (**B**) detail of an intermediate zone of the leaf blade (×400); (**C**) detail of the end of the leaf blade (×1000); (**D**) detail of an isolated vascular bundle near the end of the leaf blade (×1000); (**E**,**F**) detail of the stomata present in the epidermis (×1000). (**G**) Detailed vascular bundles facing each other and united by collenchyma (×400); (**H**) detail of a vascular bundle (×1000). Abbreviations: AbEp: Abaxial Epidermis; BS: Bundle Sheath; CC: Companion Cell; Chl: Chloroplast; Cu: Cuticle; IS: Intercellular Space; M: Mesophyll; MSc: Marginal Sclerenchyma; OC: Occlusive Cell; Ph: Phloem; Sc: Sclerenchyma; SC: Sieve-tube Cell; SStC: Substomatal Chamber; St: Stoma; TCW: Thick Cell Wall; TWMEC: Thick-Walled Marginal Epidermal Cell; V: Vacuole; VB: Vascular Bundle; WBP: Water-Bearing Parenchyma; Xy: Xylem.

The leaves are very similar in internal structure to those found in *I. aphylla* [57]. They are amphistomatic and isofacial (Figure 3A,B and Figure 4A,B), with abaxial epidermis on both sides and stomata predominantly on one side. The epidermal cells form a single layer with large, square cells (Figure 3A and Figure 4A,B). They do not have a very thick cuticle (Figure 4B,C,E,H), nor papillae or micropapillae. The partially sunken stomata are numerous and anomocytic, located transversely to the longitudinal axis of the leaf. The stomatal cells are kidney-shaped (rounded-oval in cross-section) (Figure 4E,F). The mesophyll appears isolated and spongy in cross-section (Figure 3B and Figure 4A,B,F), presenting several rows of spongy cells on both sides of the leaf, as well as an intermediate zone (Figure 3A–C and Figure 4B,G) with some air spaces and water-bearing parenchyma (Figure 3A–C and Figure 4B,G). This zone is characterized by large cells, lacking chloroplasts and thin cell walls, which allow for efficient water storage. The spongy cells of the outermost layer of the mesophyll, observed paradermally, are usually transversely elongated and parallel to epidermis (Figure 4F,G). No palisade cells are observed. At their ends, the leaves show accumulations of a V-shaped marginal sclerenchyma zone (Figure 4A,C).

Unlike *I. aphylla*, the vascular bundles occur in pairs, facing each other and close to the epidermis. They are closed and collateral. These bundles are connected by cellular cords (Figure 3A–C and Figure 4B,G). Only in areas near the ends do isolated bundles appear (Figure 4A,B). The primary elements of the phloem and xylem are clearly visible (Figure 4D,H), with characteristics typical of monocot plants.

These vascular bundles are very similar in cellular structure to those found in *I. aphylla* [57], so the primary phloem is composed almost entirely of metaphloem, with a clear difference between the sieve-tube cell and the companion cell (Figure 3D and Figure 4D,H). Primary xylem is mainly metaxylem and consists of two or three large vessels or tracheae (Figure 4D,H) with a wide lumen. In facing bundles, the vascular bundles are joined and supported by collenchyma cells (Figure 3B,C and Figure 4G). Primary xylem is mainly metaxylem and consists of two or three large vessels or tracheae (Figure 4D,H) with a wide lumen. In facing bundles, the vascular bundles are joined and supported by collenchyma cells (Figure 3B,C and Figure 4G). Above the phloem, a very evident sclerenchyma zone is observed (Figure 3B–D and Figure 4G,H), which is absent in isolated vascular bundles (Figure 4H). The bundle sheath is transparent, especially in isolated vascular bundles near the leaf ends (Figure 4H).

### 2.4. Photosynthetic Pigments

Assessing photosynthetic processes during different phenological stages in floral species involves understanding how light capture, carbon dioxide assimilation, and other physiological factors change throughout the plant life cycle, from vegetative growth to flowering and senescence [58,59]. Photosynthetic pigments in plants are responsible for light capture and energy transfer in photosynthesis and the variation in their content is an important indicator of stress caused by the environmental factors in which plants grow.

The adaptation of the species to the new growing conditions can be evidenced by analyzing the physiological processes related to the content of assimilatory pigments during the growing period. Therefore, the spectrophotometric determination of the photosynthetic pigments content was carried out in different vegetative phases: the occurrence of flowering stems at/during flowering and post-flowering.

From the analysis of the obtained results, it can be observed that in the flowering phenophase, the plants had the highest values of assimilatory pigment content, and the lowest assimilatory pigment content was obtained in the post-flowering phenophase (Table 6).

In terms of total photosynthetic pigment content, the results showed values ranging from 3.27 mg/g FW in the phenophase when the plants were in flowering to 2.90 mg/g FW in the phenophase when flowering was completed. The highest total content in photosynthetic pigments was in the flowering period of the plants (3.27 mg/g FW), the increase in value being 0.17 mg/g FW compared to the phenophase when the plants were not flowering and 0.37 mg/g FW compared to the phenophase when the plants had finished flowering.

Considering that it is emphasized in the literature that, in plants grown under normal ecophysiological conditions, the chlorophyll *a*/chlorophyll *b* ratio is 3:1 [60,61], this ratio was calculated in the study. Obtaining values of this ratio close to the one mentioned in the literature suggests that the species found optimal conditions for growth in the IULS Iasi experimental field.

From the analysis of the obtained results, it can be observed that during the flowering period the plants presented the highest value of chlorophyll *a* content (1.98 mg/g FW), which is 0.12 mg/g FW higher than the value obtained by the plants at occurrence of flowering stems and 0.33 mg/g FW higher than the value obtained by the plants post-flowering.

In the case of chlorophyll *b* content, the values maintain the same trend as in the case of chlorophyll *a* content.

Comparing the results obtained for chlorophyll *b* content with those of carotenoid pigment content, a more important increase in the values of carotenoid pigments is observed in the phenophase post-flowering of the plants (0.67 mg/g FW). Results are in agreement with studies conducted on other plant species in which abiotic stress has been shown to induce carotenoid pigment increase [62].

The highest value of the chlorophyll pigments/carotenoid pigments ratio was at plant flowering, with a value of 4.49, while the lowest value after plant flowering was 3.3. By comparing the results, it can be noticed that the obtained values are lower compared to other studies, which state that under normal ecophysiological conditions, the chlorophyll/carotenoid pigment ratio value is 4.8:1.

### 2.5. Total Phenolic and Flavonoid Contents

The total phenolic content (TPC) and total flavonoid content (TFC) were determined for methanolic (MeOH) extracts obtained from both fresh and dried leaves and roots of *I. brandzae.* A notable difference was observed between the fresh and dried samples. The highest concentrations of both phenolic and flavonoid contents were recorded in the extract from dried leaves.

**Figure 5:**
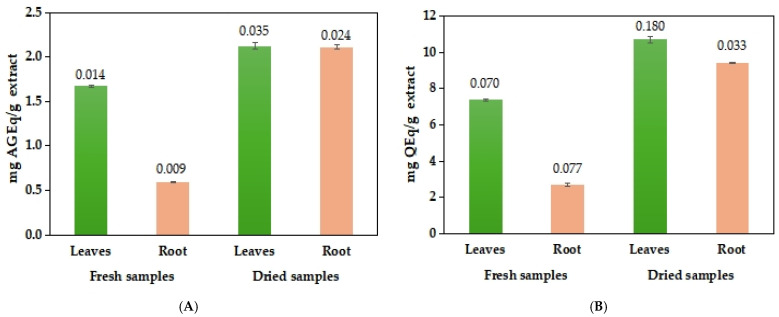
Content of polyphenols (**A**) and flavonoids (**B**) in methanolic extracts of *I. brandzae*. Error bars represent ± standard deviation (SD) of three replicates.

The TPC values ranged between 0.591 ± 0.009 and 2.126 ± 0.035 mg of gallic acid equivalents per gram of extract (mg GA Eq/g extract), with lower concentrations observed in extracts obtained from fresh tissues, as shown in Figure 5A. Regarding the TFC of methanolic extracts from *I. brandzae*, values varied between 2.70 ± 0.077 and 10.719 ± 0.180 mg of quercetin equivalents per gram of extract (mg Q Eq/g extract) for the dried leaf extract, who exhibited the most elevated TFC value, as presented in Figure 5B.

### 2.6. In Vitro-Antioxidant Evaluation

#### 2.6.1. DPPH Radical Scavenging Activity

**Figure 6:**
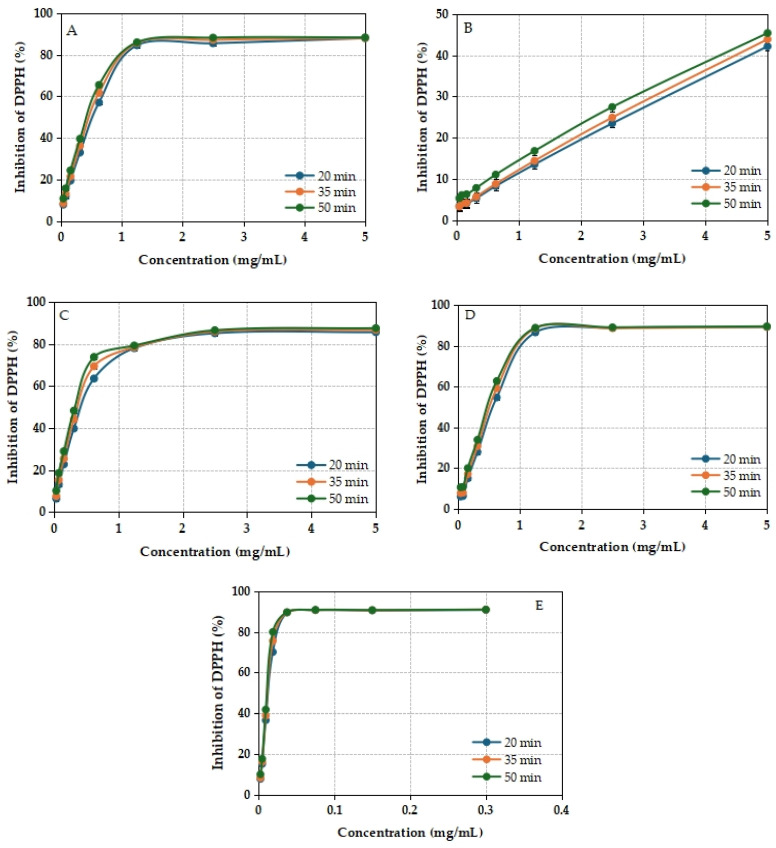
DPPH scavenging activity of methanolic extracts from fresh (**A**) leaves, (**B**) roots, dried (**C**), leaves, (**D**) roots of *I. brandzae*, and (**E**) Quercetin. Error bars represent ± standard deviation (SD) of three replicates. (Standard deviation values corresponding to the error bars are included in Tables S2 and S3).

The DPPH scavenging activity of the methanolic extracts of *I. brandzae* was evaluated in all samples. The percentage of DPPH radical inhibition remained relatively stable between 20 and 50 min of incubation, indicating a sustained antioxidant effect over time (Figure 6). The dried leaf extract showed the highest antioxidant capacity against the investigated DPPH free radicals, with an IC_50_ value of 297.1 ± 8.7140 μg/mL (Table 7). Quercetin was used as a positive control and showed a much lower IC_50_ value (10.7 ± 0.014 μg/mL) than the extracts, proving the moderate scavenging efficiency of the plant extracts. The content of phenolic compounds could justify the antioxidant activity of the alcoholic extracts of *I. brandzae*.

#### 2.6.2. ABTS Scavenging Activity

**Figure 7:**
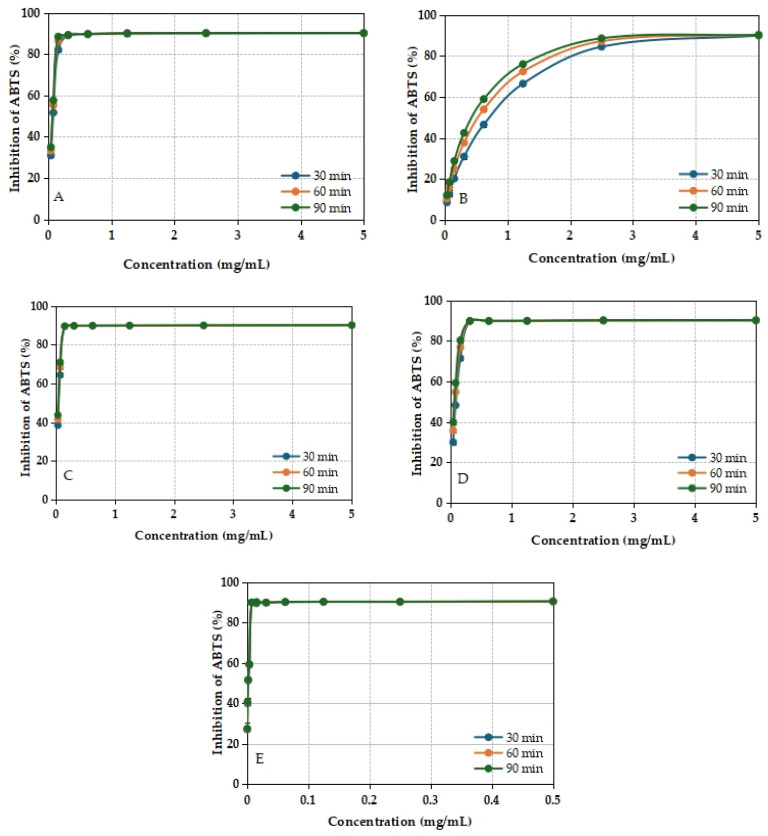
ABTS scavenging activity of methanolic extracts from fresh (**A**) leaves, (**B**) roots, dried, (**C**) leaves, and (**D**) roots of *I. brandzae* and (**E**) Trolox. Error bars represent ± standard deviation (SD) of three replicates. (Standard deviation values corresponding to the error bars are included in Tables S4 and S5).

The ABTS radical scavenging activity of the methanolic extracts of *I. brandzae* was also assessed for all analyzed samples. The percentage of ABTS radical inhibition remained stable over the incubation period, indicating a consistent antioxidant effect (Figure 7). 

Among the extracts tested, the dried leaf sample showed the strongest scavenging ability against ABTS radicals, with an IC_50_ value of 73.3 ± 0.003 μg/mL (Table 7). In comparison, the standard antioxidant Trolox exhibited a significantly lower IC_50_ value (8.3 ± 0.009 μg/mL), highlighting the moderate but notable ability of the plant extracts to neutralize free radicals. 

The total antioxidant capacity (TAC) for methanolic extracts of fresh and dried parts of *I. brandzae* confirms the previously observed patterns. The highest values of TAC were detected in dried roots (18.960 ± 0.130 mg QEq/g extract) and dried leaves (11.659 ± 0.045 mg QEq/g extract). In comparison, fresh samples showed lower TAC values; the leaves had 7.760 ± 0.071 and the root extract 6.802 ± 0.027 mg QEq/g (Table 7).

## 3. Discussion

The literature also mentions studies carried out in other species of the genus *Iris* or of the family Iridaceae in which statistical analysis based on regression models and correlations between different parameters were important methods of data interpretation. For example, to solve some taxonomic problems in phenotypically similar species within the genus *Iris*, analytical models based on morphological characters of the flower, such as the length and width of the tepals, flower length, and flower diameter, have been developed [63]. Also, in *I. lutescens*, a species with two dominant phenotypes, with yellow and purple-purple flowers, the factors that maintain flower color polymorphism within populations, i.e., their effect on flower reproductive success by color, were analyzed using data on leaf and flower size, flower stalk height, frequency of yellow and purple-flowered plants, number of plants that fruited, number of seeds formed, etc. [64].

In other species of Iridaceae, the establishment of strategies to mitigate potential losses and make the floriculture sector profitable has been based on similar study methods that have been used either in estimating the influence of ecological and technological factors on flower production and seedlings [65,66] or in analyzing trends in the production obtained and the area cultivated [67].

In wild plant conservation management, using a generalized linear model, the positive effect of mowing of mountain meadows in the Italian Alps on the cover of *G. palustris* (European protected species) and the maintenance of high vegetation diversity has been demonstrated [68]. According to the specialized literature, the anatomical characteristics of leaves play an essential role in identifying species within the genus *Iris* [69] and have significant taxonomic value in the classification of the Iridaceae family [70]. The study of the anatomical structure associated with the unfolding of physiological processes under new cultivation conditions represents a valuable tool for evaluating the behavior and development patterns of the species. Therefore, the results of studies conducted on leaf-level anatomical structures have provided valuable data regarding the anatomical characterization of the species *I. brandzae*.

Taking into account that, at present, in the specialized literature, no publications are presenting the anatomical structure at foliar level of the studied species, the results obtained were compared with the studies carried out on other species of the genus: *I. aphylla* [57], *I. magnifica* [71], *I. nezahatiae*, *I. pseudocaucasica*; *I. galatica*, *I. persica*, *I. aucheri* and *I. peshmeniana* [72], *I. korolkowii* Rege, *I. stolonifera* Maxim [73], *I. sintenisii* [74], *I. pseudacorus* and *I. sibirica* [75], and *Iris drepanophylla* [70].

The leaves of *I. brandzae* have a similar structure to those of *I. aphylla*, both species have amphistomatic and isofacial leaves with abaxial epidermis on both sides [57]. Compared to other species, the structure is different; in the case of the *I. aucheri* and *I. peshmeniana* species, the leaf is bifacial [72], and in the case of *I. pseudacorus* and *I. sibirica*, the leaf blades are of an isolateral type and covered with a cutin layer on both sides [75].

Regarding the epidermal cells, in *I. brandzae*, they are in the form of a single layer with large, square cells and do not show a very thick cuticle, papillae or micropapillae. The same characteristics of the epidermal cells, concerning the square shape and a single layer of cells, were observed in *I. peshmeniana* [72] and *I. masia* subsp. *dumaniana* [76].

In other *Iris* species, the epidermal cells show different characteristics; *I. aucheri* has upper epidermal cells that are rectangular in shape, very large, and with a single layer [72], and *I. pamphylica* shows monocellular epidermis with small, elongated cells [76]. In *I. alberti*, on the paraderm, the outline of epidermal cells is cut, rectilinear, with polygonal projection [73]; in *I. sintenisii*, cuticle covers both adaxial and abaxial surfaces of the leaf, but it is thicker on the abaxial surface, and abaxial epidermis has papillae, as observed by Akyol et al. [74].

In contrast to *I. brandzae*, papillae have been reported in numerous species: *I. aucheri*, *I. peshmeniana* [72], *I. masia* subsp. *dumaniana*, *I. masia* subsp. *masia* [76], *I. suaveolens* [74], conical papillae and micropapillae in *Iris pamphylica* [76], and dense micropapillae in *I. peshmeniana* [72], respectively.

In *I. brandzae*, the stomata present a renal shape (rounded-oval in cross-section) similar to those of *I. ahylla* [57] and *I. alberti* [73].

The mesophyll in *I. brandzae* is similar to that of *I. aphylla* [57], only of spongy type, not showing palisade parenchyma as identified in *I. sogdiana* [77], *I. pamphylica* [76], *I. aucheri* and *I. peshmeniana* [72], and *I. masia* subsp. *dumaniana* [76].

Vascular bundles in *I. brandzae* occur in pairs facing each other and close to the epidermis while in *I. aphylla,* vascular bundles are arranged in two alternating rows [57], and in *I. masia* subsp. *Dumaniana*, the vascular bundles have a single row [76].

In *I. brandzae*, above the phloem, a very conspicuous area of sclerenchyma is observed, which is absent in the isolated vascular bundles as in *I. aphylla* [57], *I. masia* subsp. *masia*, and *I. pamphylica* [76]. The xylem of the vascular bundles is oriented towards the leaf center, while the phloem is directed towards the epidermis. The primary phloem and xylem elements are clearly visible, with typical characteristics of monocotyledonous plants. In the case of some *Iris* species, the anatomical structure highlighted the presence of the bundle sheath sclerenchyma distinct from adjacent gelatinous ginder sclerenchyma, and in *I. drepanophilla*, the presence of enlarged (possibly secretory) inner bundle sheath cells [70].

Considering that the plant is cultivated under temperate continental climate conditions, it loses its foliar apparatus during the cold season when temperatures drop significantly, and the plant goes dormant. This aspect correlates with the leaf anatomy of *I. brandzae*, which did not show special adaptations specific to extreme climates (very thick cuticles, very deep stomata, abundant trichomes, very compact mesophyll, etc.).

Given that photosynthesis is a vital physiological process closely associated with plant growth and development [78,79,80], the determination of assimilatory pigment content during different phenological stages is essential for optimizing the conditions required for plant adaptation. In plants derived from wild flora, their competitive advantage in population competition and their range are indirectly influenced by the strength of photosynthetic capacity [78]. At the same time, by studying plant photosynthesis and its influencing factors, a possible optimization of environmental conditions in the applied technologies for the cultivation or propagation of plants taken from wild flora is ensured. This is considered to be an important approach in projects aimed at protecting endangered plants, restoring populations, or reintroducing them into native ranges. Growing environmental conditions, and especially hydric stress and high temperatures, reduce the photosynthetic rate per unit area of leaf surface [81], causing significant decreases in the concentration of chlorophyll *a*, chlorophyll *b*, total chlorophyll pigment content (*a* + *b*), as well as carotenoid pigment content [82]. Photosynthetic activity is related to photosynthetic pigment content [83] and photosynthetic efficiency and cell growth are associated with chlorophyll quantification [84,85].

The fluctuation in the values of the chlorophyll *a*/chlorophyll *b* ratio of 3:1 in the three phenophases confirms the results of other studies which emphasized that the values of the chlorophyll *a*/chlorophyll *b* ratio depend on the light intensity; therefore, when plants are exposed to intense light, the value of the ratio increases while shaded plants show a decrease in the ratio [57,86,87,88].

Chlorophyll *a* is considered to be not only the pigment responsible for light trapping, but also the reaction center of leaf photosynthesis, while chlorophyll *b* can act as an auxiliary pigment that can assist chlorophyll *a* in carrying out the photosynthesis process [89,90].

Since plants grow and live in dynamic environmental conditions, in which they have to respond, adapt, and acclimatize to changes in temperature, water availability, and light, the determination of chlorophyll content in plant leaves during developmental stages provides important information about the unfolding of physiological processes [91]. Determination of leaf chlorophyll *b* concentration is suitable for the study of plant resistance to environmental conditions [92]. In some *Iris* species, most of the heritability was caused by leaf chlorophyll *b* concentration, which confirms the necessity and importance of chlorophyll *b* in photosynthesis and resistance to environmental conditions [93]. Various results of chlorophyll *a* changes have been observed in numerous studies and it can be said that the response of different plant species to changes in light intensity is relatively diverse [94,95].

Carotenoid pigments exhibit dual roles, in that they act as light-trapping accessory pigments and have an essential photoprotective role by quenching excess light energy and removing ROS formed in the chloroplast [96,97]. Carotenoids are also considered to be plant pigments that function as antioxidants, hormone precursors, colorants, and essential components of the photosynthetic apparatus [98].

The effect of variation in climatic factors (especially temperature and precipitation) induces changes at the physiological level that can be evidenced by the ratio of chlorophyll pigments/carotenoid pigments [99,100].

The decrease in the chlorophyll/carotenoid pigment ratio value in the post-flowering phenophase, when the plants are preparing to enter the dormant period, suggests that *I. brandzae* plants physiologically present a stress caused by changes in abiotic factors such as temperature, light, and humidity, causing both a decrease in chlorophyll pigment content and a decrease in the chlorophyll *a*/chlorophyll *b* ratio [101,102,103].

Light controls the growth and development of plants through photosynthesis, mainly by absorbing carbon dioxide. The light intensity affecting photosynthesis varies with time and place in each habitat, although they increase their adaptation to different light intensities [104]. The maximum concentrations of photosynthetic pigments observed during flowering are directly relevant for assessing the behavior of the species *I. brandzae*, acting as bioindicators of physiological efficiency and antioxidant capacity through the photoprotective role of carotenoids. Although variations in photosynthetic pigment content may seem modest, they are biologically significant, reflecting physiological variations caused by plant adaptation to new ecological conditions, not major stress. The conceptual novelty of the study provides physiological reference parameters, essential for optimizing cultivation technologies and for conservation and reintroduction programs for this species in cultivated flora.

To complete the morphological, anatomical, and physiological studies, biochemical determinations aimed at investigating the TPC, TFC, and the antioxidant activities of methanolic extracts from *I. brandzae*. The analysis of the scientific literature did not reveal any previous research that studied the phytochemical profile of this species.

According to the literature, in the genus *Iris*, flavonoids are the most widespread class of phenolic compounds; this is also demonstrated in our analysis by the results of the total flavonoid content [3].

The plant matrix had a major influence on the content of bioactive compounds [36,105,106], fresh samples subjected to extraction presented lower results in all the performed analyses. Also, significant differences were noted, depending on the anatomical part of the plant used, and dried leaves presented the highest levels of polyphenolic compounds (2.126 ± 0.035 mg AGEq/g extract and 10.719 ± 0.180 mg QEq/g extract).

The increase in the percentage of inhibition of DPPH and ABTS free radicals depending on the concentration of the extracts suggests that the reducing capacity of the *I. brandzae* extract is comparable to that of quercetin and Trolox, respectively [107]. The exception is the methanol extract obtained from the fresh root, which at a concentration of 5 mg/mL showed a value of 45.416 ± 0.014% for DPPH radical inhibition. When evaluating the antioxidant capacity by the ABTS method, a similar result was found; the extract obtained from dried leaves had the highest antioxidant potential. The inhibition percentages of *I. brandzae* extracts and reference compounds, obtained after incubation, demonstrate that the antioxidant activity of biologically active compounds is stable over time.

The total antioxidant capacity test of *I. brandzae* extracts revealed that the dried root (18.960 ± 0.130 mg QEq/g extract) had a higher antioxidant capacity than the dried leaf (11.659 ± 0.045 mg QEq/g extract), indicating a difference between the results obtained by the DPPH and ABTS methods. This difference may be due to the mechanisms underlying each test, to act as free radical scavengers or hydrogen donors [108].

The results obtained for the evaluation of the antioxidant activity of the *I. brandzae* extracts analyzed, using the DPPH, ABTS, and TAC methods, are comparable to the data in the specialized literature, which presents plant extracts with biologically active potential from the genus *Iris* [3].

## 4. Materials and Methods

### 4.1. Description of the Site and of the Method Used to Collect the Self-Seeding Plants

**Figure 8:**
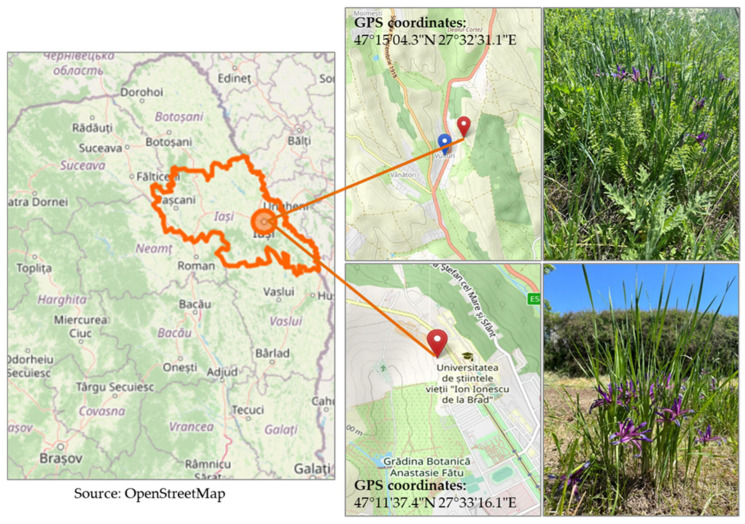
Locations of the site where *I. brandzae* was identified in the wild flora and the experimental field in which the species was cultivated.

The biological material is the *I. brandzae* species identified in Iasi County, Popricani Commune, Romania (located in the self-seeding flora of the Vulturi meadow with the following GPS coordinates 47°15′04.3″ N and 27°32′31.1″ E; Figure 8). Ten rhizome fragments were taken from this meadow in 2018, with a view to analyze the ornamental potential of the species, and were planted in the collection of ornamental plants from the self-seeding flora of Romania, located in the experimental field of the Floriculture discipline (the geographical location may be viewed using the following GPS coordinates: 47°11′37.4″ N and 27°33′16.1″ E) within the Faculty of Horticulture, Iasi University of Life Sciences (IULS), Romania. Sampling was carried out, taking into account the occurrence of the species on the meadow and all the legislative implications concerning the sozological category to which it belongs.

The species was identified in the Vulturi meadow during its flowering season, i.e., in April–May 2018, and the rhizome fragments were sampled in the post-flowering period of the plants, namely in July 2018. Our field trips enabled us to identify plants belonging to the *I. brandzae* species at various stages of development: young plants with sterile shoots (which has a single layer of leaves with more than two leaves and a poorly developed rhizome), colonies of mature plants with sterile shoots (individual plants deprived of flowers, with more than two layers of leaves, with a colony of well-developed rhizomes and with roots) and colonies of mature plants with both fertile and sterile shoots (individual plants with flowers). The biological material was collected from rhizome colonies with both sterile (vegetative) and fertile (flowering plants) shoots.

Ten rhizome fragments from ten rhizome colonies located 20 m apart from each other were sampled from the Vulturi meadow and then planted in the collection of ornamentals located in the IULS experimental field.

### 4.2. Ecological Conditions for Species Cultivation

The experimental field in which *I. brandzae* was cultivated is located in an area with an excessive temperate continental transitional climate showing aridity tendencies. The warm season is characterized by a hot and dry climate, while in the cold season, there is abundant rainfall and very low temperatures. Evaporation across the whole area is significant, with drought and aridity interrupted by heavy downpours that are often accompanied by hail, thunderstorms, and strong winds. The beginning and end of the winter season are usually characterized by early and late frost, fog, and snowfalls caused by horizontal movements of cold and very cold air of polar or arctic origin.

The soil on which the plants were grown in the floricultural collection of IULS has a sandy texture with a high content of fine gravel, typical of well-drained substrates. The granulometric analysis revealed a large share of coarse particles (>1 mm−65%) and a low content of dust and clay (3.41%), which encompasses low water-holding capacity and very good aeration. As far as its chemical composition is concerned, the soil is weakly alkaline (pH 7.8) with a specific electrical conductivity of 0.152 mS/cm. The organic carbon (2.35%) and mineral nitrogen (NO_3_^−^ + NO_2_^−^ + NH_4_^+^ = 17.6 ppm) content indicate moderate organic fertility. The concentrations of macroelements accessible to plants are, however, low: 17.3 ppm P, 10 ppm K, 16.2 ppm Na, 73.4 ppm Ca, and 6.2 ppm Mg. In terms of microelements, the soil shows moderate to good contents: manganese (16.8 ppm) and zinc (5.8 ppm), copper (7.9 ppm) and iron (13.1 ppm), although the weak alkaline reaction may partially reduce the availability of some ions, especially iron and manganese.

In Table S1 we focus on the main meteorological parameters during the six years of experimental studies, e.g., temperature, precipitation, and sunlight duration. The average annual temperature was 11.46 °C, with extremes ranging from −2.9 °C in winter to 23.5 °C in summer. Significant variations in temperature were observed in January 2023, with an average of 2.8 °C in January (approx. 5 °C higher than January 2019). February 2023 remained consistent with 2019 values (1.6 °C vs. 1.8 °C). We noted a slight increase in the annual mean temperature in 2020 with 0.80 °C. During the flowering season (April–May), the highest temperatures were recorded in 2020 and 2022, with deviations from the mean of 1.74 °C and 1.12 °C, respectively. The mean temperature evolution showed large differences from the normal mean in March 2019, 2020, and 2023.

In 2022, the growing season started with very low rainfall in March (8.20 mm); this is the period in which the *I. brandzae* species starts vegetation. When observing the mean annual precipitation readings, we can notice that 2022 was the year with the lowest annual rainfall (416.8 mm) across the six-year study. The years 2018 and 2020 were characterized by very large variations, marked by significant declines in April and substantial increases in July relative to the same months in other years.

Throughout the study period, the mean monthly sunshine duration showed large fluctuations from year to year, primarily due to reduced insolation during the cold season. Despite the variations recorded, both within each calendar year, between the experimental years, the annual sum of sunshine duration was not very different.

### 4.3. Study of Morphological Characteristics

The biometric measurements were carried out over five years during the growing season, from the plants’ vegetation onset in March 2019 (8 months after crop establishment) until the plants entered dormancy in October 2023. The morphological characteristics were studied in 50 plants, of which 25 did not form flowers and were considered as sterile shoots, while the remaining 25 formed flowers and were considered as fertile shoots. The study of the morphological characteristics included biometric indicators for sterile shoots: number of leaves (piece—pc), leaf length (cm), and maximum leaf width (cm). For fertile shoots, the following parameters were recorded: number of flowers per stem (pc), flowering stem length (cm), number of leaves (pc), leaf length (cm), maximum leaf width, number of bracts per stem (pc) and bract length (cm) as 01, 02, 03, and 04. Measurements of the four protective bracts (spathe), arranged as two per flower, were sequentially numbered from the stem base to the apex morphological traits were taken with a ruler graduated in millimeters, as commonly applied in field studies. All findings of the biometric indicators were statistically processed using multiple linear regression and a one-way ANOVA test, in order to gain a detailed understanding of the influence of each parameter on the dependent variable and to determine whether there are significant differences among the means of three or more independent groups. In addition, exploratory multilinear regression models were tested, such as using flowering stem height as the dependent variable and perianth-segments or bract (spathe) dimensions as predictors. These additional models did not reveal statistically significant relationships (*p* > 0.05).

### 4.4. Anatomical Study

Anatomical studies on leaf structure were conducted using two techniques: (1) the tissue freezing technique and (2) the resin embedding technique. Leaves from the same position were randomly collected from each of the 10 individual plants to ensure consistency. In order to make sections by the tissue freezing technique, ten samples (leaves) were cut into 20–30 µm thick sections using a freezing microtome (CM 1325; Leica, Wetzlar, Germany). The obtained sections were stained for 5 min with FSA (Basic Fuchsin, Safranin, and Astra Blue), then rinsed in water and mounted for analysis. The sections were analyzed and photographs were taken using a set of equipment consisting of an optical microscope (OLYMPUS BX50, Tokyo, Japan) equipped with an Axiocam 208 digital color camera and analyzed with ZEN 3.0 software (Carl Zeiss Microscopy GmbH, Munich, Germany). When anatomical structures were obtained using the resin embedding technique, another set of ten leaf samples was fixed in FAA (formaldehyde, alcohol, acetic acid), rinsed in three steps for 15 min each with 0.01 M PBS (phosphate-buffered saline), pH 7.4. The last step was dehydration of the samples at room temperature, by immersing them in a graded sequence of ethanol: 50%, 70%, 95%, 100%, for 20–30 min for each concentration.

In order to obtain resin blocks with samples fixed and dehydrated in Spurr resin, we followed the steps suggested by the manufacturer’s protocol [109]. Optical microscopic analysis was performed on 1-2 µm sections of the samples embedded in Spurr resin and cut with a diamond knife (DIATOME Histo 45) attached to an ultramicrotome (Ultratome Nova LKB Bromma, Stockholm, Sweden). Images were acquired by staining the sections with toluidine blue 1% and viewing them with an OLYMPUS BX50 optical microscope equipped with an Axiocam 208 digital camera. Images of anatomical structures were captured and analyzed using ZEN 3.0 software (Carl Zeiss Microscopy GmbH, Germany).

### 4.5. Determination of Photosynthetic Pigment Content

The determination of photosynthetic pigment content was carried out before the occurrence of the flowering stem, during the flowering period and after the end of flowering. Fully developed leaves were randomly harvested from the same position on 10 individual plants, then homogenized, and three samples were prepared in triplicate to obtain material for pigment extraction.

The extracts necessary for the analysis of photosynthetic pigments in *I. brandzae* leaves were obtained using the method described by Lichtenthaler and Buschmann [88]. The analysis of photosynthetic pigments was carried out by spectrophotometric means, using the UV-VIS spectrophotometer (T70 UV/VIS Spectrophotometer, PG Instruments Ltd., Wibtoft, Leicestershire, UK). For each vegetation phenophase, 0.03-0.05 g of fresh leaf tissue per sample was used, with all samples being analyzed in triplicate; all data are reported as mean ± standard deviation.

The tissue was continuously milled by adding 2–3 mL of pure acetone until complete homogenization of the plant material. Subsequently, the resulting extract was transferred into a graduated cylinder, and the process was repeated until a colorless filtrate was obtained.

The procedure was repeated until the volume of the filtrate reached 10 mL, which was then centrifuged at 10,000× *g* for 10 min. The photosynthetic pigment content was determined by reading the extract previously obtained at wavelengths of 661.6 nm for chlorophyll a, 644.8 nm for chlorophyll b, and 470 nm for carotenoid pigments [88].

All assimilatory pigment content determinations were performed at the Horticultural Research Center of the Faculty of Horticulture, IULS, Romania.

### 4.6. Ultrasound-Assisted Extraction with Methanol

The fresh and dried leaves and roots of *I. brandzae* were used for the extraction of phytochemical constituents, following the procedure described by Busuioc et al. [110]. For the analysis, 10 mg of the dry extract was redissolved in methanol. All measurements were performed in triplicate, as described above for pigment determination, and all data are expressed as the mean ± standard deviation.

#### 4.6.1. Determination of Total Phenolic Content

The total phenolic content was analyzed using the Folin–Ciocalteu colorimetric method. Gallic acid was used as a reference standard and the results were expressed as milligram gallic acid equivalents per gram of extract (mg GA Eq/g extract).

#### 4.6.2. Determination of Total Flavonoid Content

The total flavonoid content was determined according to the literature using an aluminum chloride colorimetric assay [111]. The results were expressed as milligram quercetin equivalents per g of extract (mg Q Eq/g extract), determined from a calibration curve prepared with quercetin. Each sample was analyzed in triplicate, and all data are reported as mean ± standard deviation.

#### 4.6.3. In Vitro-Antioxidant Assays

In the present study, the antioxidant potential of the methanolic extracts from *I. brandzae* was assessed using three methods published in previous studies [110,112,113].

The DPPH (2,2-diphenyl-1-picrylhydrazyl) radical scavenging of the extracts was evaluated as previously described by Daraban et al. [113]. Briefly, 100 μL of each extract (0.04–5 mg/mL) was mixed with 100 µL DPPH solution and incubated in the dark at room temperature for 50 min and the absorbance was then measured at 517 nm with a microplate reader with 96-well plates. Quercetin was used as a positive control. The percentage of DPPH inhibition was calculated using the following Equation (1):

(1) $$Inhibition\;of\;DPPH\;\left(\%\right)=\;\frac{A_{control}-A_{sample}}{A_{control}}\times 100$$

where *A_control_* represents the absorbance of the control, and the *A_sample_* represents the absorbance of the sample.

The ABTS (2,2-Azino-bis (3-ethylbenzothiazoline-6-sulfonic acid)) radical scavenging activity was carried out following the protocol by mixing 100 µL of each extract (0.04–5 mg/mL) with 100 µL of ABTS solution and the absorbance was recorded at 734 nm after incubation in the dark, using a 96-well microplate at specific time intervals (30, 60, and 90 min). Trolox was used as a positive control. The percentage of ABTS inhibition was determined using the formula presented below (2):

(2) $$Inhibition\;of\;ABTS\;\left(\%\right)=\;\frac{A_{control}-A_{sample}}{A_{control}}\times 100$$

where *A_control_* represents the absorbance of the control, and the *A_sample_* represents the absorbance of the sample.

The half maximal inhibitory concentrations (IC_50_) for both DPPH and ABTS assays were obtained from the curve illustrating the relationship between radical scavenging capacity and the corresponding sample concentrations. Lower IC_50_ values indicate stronger antioxidant potential, representing the concentration required to reduce the initial free radical concentration by 50%.

The total antioxidant capacity (TAC) was evaluated with the phosphomolybdenum method, as described by Zongo et al. [112]. Briefly, 0.1 mL of methanolic extract (5 mg/mL concentration) was mixed with 1 mL of reagent solution, incubated for 90 min at 95 °C. After cooling, absorbance was measured at 695 nm and results were expressed as milligram quercetin equivalents per g of extract (mg Q Eq/g extract).

## 5. Conclusions

The results of the study provide new information about the *I. brandzae* species, thus supplementing the existing literature focused solely on the botanical description, distribution, ecology, and biogeography of the species with anatomical, physiological, and biochemical data that had not been studied until now. Therefore, our research aimed at using anatomical–morphological, physiological, and biochemical biomarkers to evaluate the behavior in new ecological conditions, the ornamental potential, and the antioxidant capacity of a wild species of *I. brandzae*.

The Pearson correlation coefficient between leaf length and leaf width indicates a moderate positive linear association, suggesting that, on average, longer leaves tend to be wider. The linear regression model obtained between leaf length and leaf width indicated that about 44% of the variation in leaf width can be explained by the variation in leaf length. Application of the one-way ANOVA test revealed significant differences between group means for bract length.

The total content of photosynthetic pigments showed maximum values during the flowering period (3.27 mg/g FW) and minimum values in the post-flowering period (2.90 mg/g FW). The chlorophyll a/b ratio ranged from 2.96 to 2.84, and the chlorophyll/carotenoid ratio ranged from 4.60 to 3.0, indicating increased photosynthetic activity during flowering.

The anatomical structure of *I. brandzae* leaves highlights the species’ behavior in new cultivation conditions, by showing epidermal cells in a single layer without thick cuticle, papillae, or micropapillae; anomocytic stomata was arranged transversely and spongy mesophyll bilaterally. The arrangement of vascular bundles in pairs, where the xylem is centrally oriented with the phloem towards the epidermis, highlights characteristics typical of monocotyledons, supporting the biological adaptation potential of the species.

The total antioxidant capacity test of *I. brandzae* extracts revealed that the dried root had a higher antioxidant capacity than the dried leaf; this difference may be due to the mechanisms underlying each test, to act as free radical scavengers or hydrogen donors. The results obtained for the evaluation of the antioxidant activity of the *I. brandzae* extracts analyzed are comparable to the data in the specialized literature of the genus *Iris* and highlight that the studied species has antioxidant capacity.

Based on the results obtained, future research should focus on comparative studies of morphological, anatomical, physiological, and biochemical parameters in plants from the experimental field and from their natural habitat. Additional investigations on seasonal variation in pigment content, correlations between phytochemical composition and environmental factors, and adaptive responses under controlled stress conditions would further contribute to understanding the ecological behavior of *I. brandzae*.

The ornamental value of the species, early flowering, behavior in other ecological conditions, and antioxidant potential are considered important indicators that recommend this species as valuable not only for enriching the floral assortment, but also for inclusion in breeding programs.

## Figures and Tables

**Table 1:** Mean values of leaf dimensions (cm) by shoot types.

	Sterile Shoots		Fertile Shoots	
	Leaf Length	Leaf Width	Leaf Length	Leaf Width
Mean	50.5	0.45	18.40	0.37
CI (confidence level 95%)	[47.82, 53.29]	[0.43, 0.46]	[16.46, 20.33]	[0.35, 0.39]

**Table 2:** Mean values of perianth-segments dimensions and flowering stem height (cm).

	Flowering Stem Height	Outer Perianth-Segments		Inner Perianth-Segments	
		Length	Width	Length	Width
Mean	27.3	4.18	0.85	2.70	0.64
CI (confidence level 95%)	[24.60, 30.02]	[4.08, 4.28]	[0.78, 0.92]	[2.59, 2.82]	[0.58, 0.71]

**Table 3:** Mean values of morphological characteristics of fertile shoots.

	Flowering Stem Height (cm)	No. Leaves/Shoot (no.)	Bract Length 01 (cm)	Bract Length 02 (cm)	Bract Length 03 (cm)	Bract Length 04 (cm)
Mean	27.31	2.83	8.68	6.25	8.02	7.17
CI (confidence level 95%)	[24.60, 30.02]	[2.47, 3.20]	[8.09, 9.26]	[5.68, 6.64]	[7.46, 8.57]	[6.64, 7.69]

**Table 4:** The one-way ANOVA test.

Source of Variation	SS	df	MS	F	*p*-Value
Between Groups	39.81895833	3	13.2729861	19.894219	2.681 × 10^−8^
Within Groups	29.35583333	44	0.66717803	-	-
Total	69.17479167	47	-	-	-

**Table 5:** *p*-values following Student’s *t*-test.

	Bract Length 02	Bract Length 03	Bract Length 04
Bract length 01	0.0000347	0.0850000	0.0003420
Bract length 02		0.0093750	0.0054800
Bract length 03			0.0229000

Level of confidence 0.05.

**Table 6:** Average photosynthetic pigment content in *Iris brandzae*.

Vegetation Phenophase	Chl. *a* mg/g FW	Chl. *b* mg/g FW	Car mg/g FW	Σ	Chl. *a*/ Chl. *b*	Chl./Car.
The occurrence of flowering stems	1.86 ± 0.04	0.64 ± 0.03	0.60 ± 0.03	3.10	2.91	4.17
At/during flowering	1.98 ± 0.03	0.67 ± 0.02	0.62 ± 0.02	3.27	2.96	4.49
Post-flowering	1.65 ± 0.02	0.58 ± 0.04	0.67 ± 0.04	2.90	2.84	3.33

Each value is shown as the mean ± S.D.; FW—fresh weight; Chl. *a*—chlorophyll *a*; Chl. *b*—chlorophyll *b*; Car.—carotenoids.

**Table 7:** Antioxidant activity of methanolic extracts of *I. brandzae* determined by DPPH, ABTS, and TAC assays.

Material	Vegetative Organs	DPPH (IC_50_ μg/mL)	ABTS (IC_50_ μg/mL)	TAC (mg QEq/g Extract)
Fresh samples	Leaves	412.6 ± 6.421	82.6 ± 1.510	7.760 ± 0.071
	Root	13,779.2 ± 13.760	463 ± 1.806	6.802 ± 0.027
Dried samples	Leaves	297.1 ± 8.714	73.3 ± 3.058	11.659 ± 0.045
	Root	462.8 ± 5.985	83.9 ± 2.344	18.960 ± 0.130
Quercetin		10.7 ± 0.014	-	-
Trolox		-	3 ± 0.059	-

Each value is shown as the mean ± S.D.

## Data Availability

The original contributions presented in this study are included in the article/Supplementary Materials. Further inquiries can be directed to the corresponding authors.

## Supplementary Materials

The following supporting information can be downloaded at: https://www.mdpi.com/article/10.3390/plants14243803/s1. Table S1: Meteorological parameters in the field (2018–2023); Table S2: Standard deviation (SD) results from the DPPH scavenging activity of methanolic extracts from fresh leaves and roots, dried leaves and roots; Table S3: Standard deviation (SD) results from the DPPH scavenging activity of Quercetin; Table S4: Standard deviation (SD) results from the ABTS scavenging activity of methanolic extracts from fresh leaves and roots, dried leaves and roots; Table S5: Standard deviation (SD) results from the ABTS scavenging activity of Trolox.
