# Supplementary material for: Initial Characterization of Morpho-Anatomical Traits and Antioxidant Profile of Iris brandzae Prodan from Romania’s Wild Flora Under Culture Conditions
Source: Plants (Basel). 2025 Dec 13;14(24):3803. doi: 10.3390/plants14243803 (PMC12737319; doi:10.3390/plants14243803)
Supplement: Supplementary file 1 [file plants-14-03803-s001.zip › plants-3995244-supplementary.pdf]

plants

MDPI

Supplementary material

Initial Characterization of Morpho-Anatomical Traits and Antioxidant Profile of *Iris brandzae* Prodan from Romania's Wild Flora under Culture Conditions

Lucia Draghia [1], Maria Apostol [1,*], Culiță Sîrbu [1], Ivayla Dincheva [2], Maria Daniela Mihăilă Ionică [3], Rodica Mihaela Dinică [3], Mariana Lupoae [4,5], Raluca-Maria Hlihor [1], Isabela Maria Simion [1], Ciprian Chiruță [1], Diana Elena Bolohan [1], Jose Reig Arminana [6] and Francisco José Garcia Breijo [7,*]

[1] "Ion Ionescu de la Brad" Iasi University of Life Sciences, 3 Mihail Sadoveanu Alley, 700490 Iasi, Romania; lucia.draghia@iuls.ro (L.D.); culita.sirbu@iuls.ro (C.S.); raluca.hlihor@iuls.ro (R.-M.H.); isabela.simion@iuls.ro (I.M.S.); ciprian.chiruta@iuls.ro (C.C.); diana.bolohan@iuls.ro (D.E.B.)
[2] Department of Agrobiotechnologies, Agrobioinstitute, Agricultural Academy, 8 Dragan Tsankov Blvd, Sofia, 1164, Bulgaria; ivadincheva@yahoo.com (I.D.)
[3] Department of Chemistry, Physics and Environment, Faculty of Sciences and Environment, "Dunarea de Jos" University of Galati, 111 Domneasca Street, 800201 Galati, Romania; maria.mihaila@ugal.ro (M.D.M.I.); rodinica@ugal.ro (R.M.D.)
[4] Department of Pharmaceutical Sciences, Faculty of Medicine and Pharmacy, "Dunarea de Jos" University of Galati, 35 Alexandru Ioan Cuza Street, 800010 Galati, Romania; mariana.lupoae@ugal.ro (M.L.)
[5] Medical-Pharmaceutical Research Center, Faculty of Medicine and Pharmacy, "Dunarea de Jos" University of Galati, 35 Alexandru Ioan Cuza Street, 800010 Galati, Romania
[6] Instituto Cavanilles de Biodiversidad y Biología Evolutiva, Universitad de València, 46980 Valencia, Spain; jose.reig@uv.es (J.R.A.)
[7] Departamento de Ecosistemas Agroforestales, Escuela Técnica Superior de Ingeniería Agronómica y del Medio Natural (ETSIAMN), Universitat Politècnica de València, Camino de Vera s/n., 46022 Valencia, Spain
* Correspondence: maria.apostol@iuls.ro (M.A.); fjgarci@eaf.upv.es (F.J.G.B.)

Table S1. Meteorological parameters in the field (2018–2023).

**Average monthly temperatures (°C)**													
**Months/Years**	**I**	**II**	**III**	**IV**	**V**	**VI**	**VII**	**VIII**	**IX**	**X**	**XI**	**XII**	**Means**
**2018**	−1	−2.2	0.8	15.3	19.1	20.7	21.2	22.9	16.7	12.3	2.5	−1.4	**10.5**
**2019**	-2.9	1.8	7.1	10.4	16	22.4	21.5	22.5	17.4	11.4	8.1	3.2	**11.5**
**2020**	1.1	4.3	7	11.3	14	20.9	22.7	23.5	19.6	13.6	4.5	1.8	**12.3**
**2021**	0.1	-0.9	3.1	8.1	15.4	19.7	23.4	20.9	14.7	9.5	6.7	0.2	**10.0**
**2022**	0.3	3.7	3.0	9.9	16.8	21.9	23.2	22.5	15.5	12.5	5.5	1.4	**11.3**
**2023**	2.8	1.6	6.6	8.1	16.2	20.4	23.0	24.6	19.9	14.4	6.6	2.7	**12.2**
**Monthly precipitations (mm)**													
**Months/Years**	**I**	**II**	**III**	**IV**	**V**	**VI**	**VII**	**VIII**	**IX**	**X**	**XI**	**XII**	**Sum**
**2018**	38.8	37.0	72.2	9.2	13.6	219.6	184.2	3.0	30.4	2.6	64.6	52.6	**727.8**
**2019**	50.6	32.8	9.8	46	98.6	63	33.8	43.2	38.8	30.6	10.2	21.3	**478.7**
**2020**	3.6	43.2	18.2	8.4	102.2	108.4	42	9.2	29.8	104.8	22.8	54.8	**547.4**
**2021**	28.4	24.6	50.4	53.2	68.2	93.6	87.6	95.4	10.4	2.8	8.8	69,2	**593.0**
**2022**	4.8	10.0	8.2	73.6	29.2	26.6	27.8	69	69.6	12.6	69.2	16.2	**416.8**
**2023**	12.1	23.3	5.8	157.7	25.6	33.2	107.8	16.4	8.6	16.2	92.4	10.0	**509.1**
**Sunlight duration (hours)**													

Months/Years	I	II	III	IV	V	VI	VII	VIII	IX	X	XI	XII	Sum
**2018**	53.8	57.5	214	192.7	328.5	231.2	214.1	284.8	252.7	140.8	64.2	93.5	**2127.8**
**2019**	83.1	72.5	151.1	239.2	285.5	282.8	276.5	198.4	199.9	190.6	96.4	73.6	**2149.6**
**2020**	99.6	116.4	191	279.8	178.2	235.7	275.6	295.3	259.5	123.1	65.1	25.5	**2144.8**
**2021**	65.2	107.1	163.4	183.7	212.8	218	283.7	265.1	188.3	195.9	116.4	30.1	**2029.7**
**2022**	79.3	112.5	161.4	167.7	283.9	262.6	273.7	225.8	155.4	188.6	56.6	64.9	**2032.4**
**2023**	49.9	107.9	180.6	120.4	235.3	242.2	285.2	294.0	240.7	180.9	81.4	62.6	**2081.1**

Table S2. Standard deviation (SD) results from the DPPH scavenging activity of methanolic extracts from fresh leaves and roots, dried leaves and roots.

Fresh samples								Dried samples							
Leaves				Root				Leaves				Root			
mg/mL	20 min	35 min	50 min	mg/mL	20 min	35 min	50 min	mg/mL	20 min	35 min	50 min	mg/mL	20 min	35 min	50 min
5.00	0.950	0.941	0.986	5	0.989	0.624	0.390	5	0.284	0.280	0.326	5	0.188	0.229	0.310
2.50	0.032	0.046	0.041	2.5	0.197	0.145	0.185	2.5	0.206	0.249	0.311	2.5	0.120	0.130	0.106
1.25	0.450	0.280	0.142	1.25	0.488	0.490	0.477	1.25	0.046	0.147	0.201	1.25	0.335	0.229	0.230
0.63	0.574	0.501	0.472	0.625	0.774	0.716	0.674	0.625	0.211	0.180	0.165	0.625	0.118	0.160	0.156
0.31	0.459	0.474	0.416	0.3125	0.282	0.344	0.267	0.3125	0.159	0.561	0.972	0.3125	0.247	0.207	0.156
0.16	0.582	0.674	0.624	0.15625	0.622	0.741	0.737	0.15625	0.927	0.976	0.917	0.15625	0.495	0.590	0.675
0.08	0.870	0.159	0.943	0.078125	0.277	0.365	0.405	0.078125	0.497	0.987	0.606	0.078125	0.707	0.604	0.581
0.04	0.198	0.162	0.076	0.0390625	0.750	0.852	0.938	0.0390625	0.844	0.853	0.791	0.0390625	0.345	0.307	0.671

Table S3. Standard deviation (SD) results from the DPPH scavenging activity of Quercetin.

ug/mL	20 min	35 min	50 min
0.3	0.183	0.164	0.199
0.15	0.168	0.121	0.141
0.075	0.183	0.201	0.184
0.0375	0.045	0.041	0.054
0.01875	0.621	0.614	0.420
0.009375	0.535	0.635	0.795
0.0046875	0.910	0.900	0.981
0.00234375	0.966	0.909	0.945

Table S4. Standard deviation (SD) results from the ABTS scavenging activity of methanolic extracts from fresh leaves and roots, dried leaves and roots.

Fresh samples								Dried samples							
Leaves				Root				Leaves				Root			
mg/mL	20 min	35 min	50 min	mg/mL	20 min	35 min	50 min	mg/mL	20 min	35 min	50 min	mg/mL	20 min	35 min	50 min
5	0.131	0.198	0.262	5	0.502	0.292	0.294	5	0.212	0.273	0.316	5	0.228	0.126	0.235
2.5	0.258	0.257	0.296	2.5	0.296	0.286	0.279	2.5	0.323	0.260	0.191	2.5	0.214	0.475	0.241
1.25	0.324	0.325	0.310	1.25	0.188	0.207	0.228	1.25	0.251	0.249	0.290	1.25	0.335	0.075	0.289
0.625	0.152	0.051	0.051	0.625	0.474	0.507	0.495	0.625	0.038	0.043	0.042	0.625	0.190	0.162	0.211
0.3125	0.076	0.062	0.049	0.3125	0.203	0.210	0.279	0.3125	0.498	0.546	0.552	0.3125	0.382	0.215	0.313
0.15625	0.794	0.438	0.292	0.15625	0.466	0.434	0.466	0.15625	0.138	0.136	0.150	0.15625	0.780	0.560	1.042
0.078125	0.485	0.431	0.534	0.078125	0.716	0.711	0.667	0.078125	0.953	0.969	0.966	0.078125	0.824	0.834	0.799
0.0390625	0.999	0.922	0.917	0.0390625	0.740	0.839	0.833	0.0390625	1.032	1.096	1.097	0.0390625	1.031	1.063	1.046

Table S5. Standard deviation (SD) results from the ABTS scavenging activity of Trolox.

ug/mL	30 min	60 min	90 min
0.5	0.150	0.151	0.208
0.25	0.335	0.321	0.319
0.125	0.310	0.280	0.312
0.0625	0.169	0.144	0.161
0.03125	0.282	0.270	0.340
0.015625	0.645	0.301	0.257
0.0078125	0.162	0.136	0.136
0.00390625	0.825	0.837	0.823
0.001953125	1.591	1.275	1.059
0.000976563	2.503	2.110	2.898
0.000488281	3.525	3.392	3.243
